# Surface-Controlled Sialoside-Based Biosensing of Viral and Bacterial Neuraminidases

**DOI:** 10.1021/acs.langmuir.3c03943

**Published:** 2024-03-30

**Authors:** Israel Alshanski, Suraj Toraskar, Daniel Gordon-Levitan, Marco Massetti, Prashant Jain, Luigi Vaccaro, Raghavendra Kikkeri, Mattan Hurevich, Shlomo Yitzchaik

**Affiliations:** † The Institute of Chemistry and Center of Nanotechnology, The Hebrew University of Jerusalem, Jerusalem 91904, Israel; ‡ Indian Institute of Science Education and Research, Dr. Homi Bhabha Road, Pune 411008, India; § Laboratory of Green Synthetic Organic Chemistry, Dipartimento di Chimica, Biologiae Biotecnologie Università di Perugia, Via Elce di Sotto 8, 06123 Perugia, Italy

## Abstract

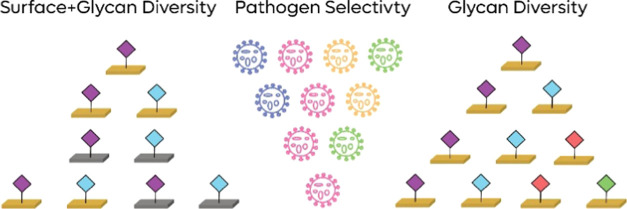

Neuraminidases (NA) are sialic acid-cleaving enzymes that are used by both bacteria and viruses. These enzymes have sialoside structure-related binding and cleaving preferences. Differentiating between these enzymes requires using a large array of hard-to-access sialosides. In this work, we used electrochemical impedimetric biosensing to differentiate among several pathogene-related NAs. We used a limited set of sialosides and tailored the surface properties. Various sialosides were grafted on two different surfaces with unique properties. Electrografting on glassy carbon electrodes provided low-density sialoside-functionalized surfaces with a hydrophobic submonolayer. A two-step assembly on gold electrodes provided a denser sialoside layer on a negatively charged submonolayer. The synthesis of each sialoside required dozens of laborious steps. Utilizing the unique protein–electrode interaction modes resulted in richer biodata without increasing the synthetic load. These principles allowed for profiling NAs and determining the efficacy of various antiviral inhibitors.

## Introduction

**Figure 1:**
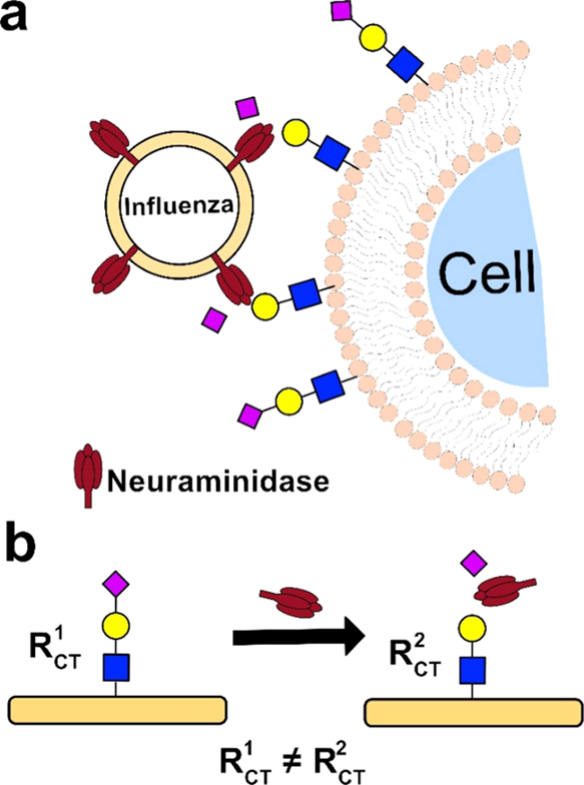
(a) Influenza utilizes NA to detach from the cell by cleaving sialic acid (pink diamond) from cell surface sialosides. (b) NA cleaves sialic acid from a sensory layer interface on an electrode to produce an electrochemical signal (*R*_CT_, charge-transfer resistance).

Glycans decorated with sialic acid, sialosides, serve for communication between cells, recognition, and immune activity.^1−3^ Sialosides are commonly found at cellular interfaces. Sialosides differ in their monosaccharide type, the connectivity to the core glycan, and the glycan types themselves, e.g., *O*-glycans or *N*-glycans. The composition of the cell surface sialosides is influenced by the tissue type, environmental factors, and pathological states. Viral and bacterial pathogens bind and hydrolyze sialic acid.^4,5^ Influenza virus (IV) is a common infectious pathogen that can cause serious inflammation and even death and might lead to a pandemic.^6^ IV targets sialic acid via two protein families, namely, hemagglutinins (HAs) and neuraminidases (NAs).^6−8^ HA binds sialosides, while NA cleaves sialic acid from the cell surface (Figure 1a).^9^ IV strains differ in their HA and NA components, which dictates their preferred sialoside binding and catalysis.^10^ Therefore, influenza strains can be characterized by sialoside profiling.^7^ Early and accurate detection of IV strain is crucial for determining the treatment strategy. Sialoside-based IV NA profiling can provide this valuable information.

Electrochemical impedance spectroscopy (EIS) is a label-free analytical technique that can be used for the detection of ions, biomolecules, proteins, and even cells.^11−16^ The interaction of a recognition layer with an analyte causes changes in its dielectric properties. In EIS, these changes translate into a measurable resistance and capacitance signal.^17−19^ EIS principles enable selective and sensitive detection of IV proteins.^20,21^ Biosensors that can target proteins are generally composed of electrodes grafted with a biomolecular recognition layer, e.g., glycans or peptides. Interaction of the recognition layer with the protein analyte, resulting in either binding or enzymatic activity, induces morphological changes in the electrode surface. The electrochemical signal for protein binding originates from the adhesion of a large moiety to the surface. Enzymatic activity acts on the molecular level, thus enabling protein detection through conformational changes in the recognition layer (Figure 1b). Protein electrochemical biosensing relies on the interactions of the binding site with the recognition moiety and is also affected by peripheral interaction surrounding the active sites on the protein and the electrode.^13,22^ These peripheral interactions are determined by the surface charge, density, and other weak interactions around the primary binding site.^11,19,23^

Designing a sensing system that considers both the recognition moiety and the surface properties can enable selectivity that cannot be obtained by focusing on only the sensing moiety. Since IV NAs interact with many different sialosides preferentially, they cannot be accurately analyzed by a single moiety biosensing strategy. Using biosensor array and principle component analyses proved to be useful in discriminating between undistinguishable analytes.^10,22,24,25^ Employing the sensitive EIS methodology for the detection of NAs using a sialoside array grafted on variable surfaces can enable selective determination of IV NA type and set the ground for the IV NA inhibitor efficacy evaluation strategy.^23,26,27^

**Figure 2:**
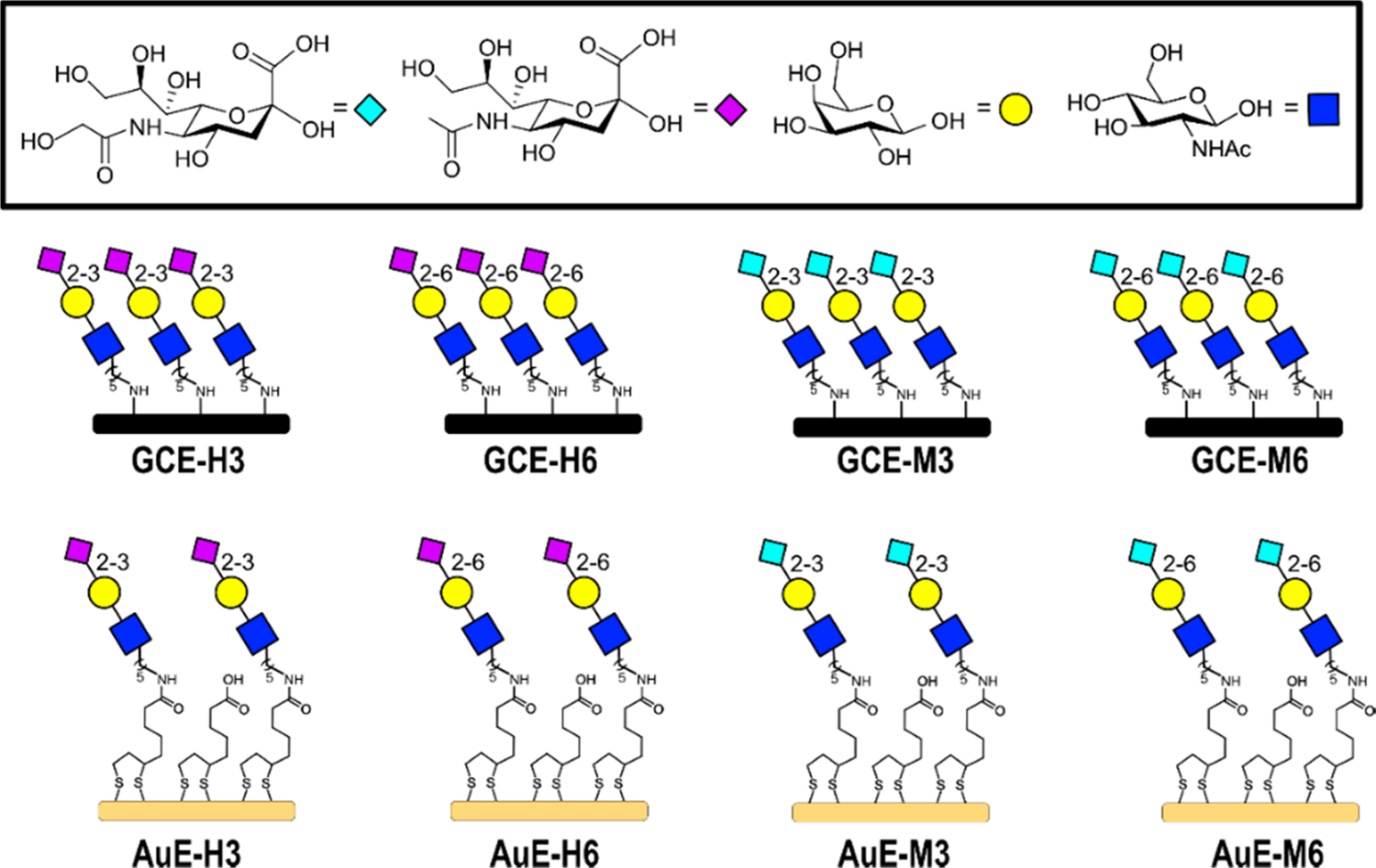
Modified glassy carbon electrode (GCE) layers, which were formed as hydrophobic glycan-modified layers by electrodeposition of aminyl trisaccharides, and AuE layers, which were formed as negatively charged glycan-modified layers by amidation of aminyl trisaccharides with lipoic acid (LPA), that were used in this work (see Schemes S1 and S2 for full structure examples of GCE-H3 and AuE-H3, respectively).

Previously, we showed that by using the same sialosides on different electrodes, we can discriminate between binding and catalysis of bacterial NAs.^23^ Herein, we harnessed the developed strategy for impedimetric detection of various types of IV NAs by collective evaluation of binding and catalytic activity of the sialoside array on two types of interfaces (Figure 2). We aim to use EIS to profile IV NAs through a combination of sialoside library and electrode surface features. This also enabled the discrimination of bacteria to IV NAs and assess inhibitor efficacy.

## Experimental Section

### Materials

All reagents were purchased from Sigma-Aldrich (Merck), including inhibitors. Recombinant *H1N1*, *H5N1*, and *H3N2* neuraminidases were purchased from Bio-Techne.

### Preparation of Modified Glassy Carbon Electrodes

GCEs were manually polished on a microcloth pad (Buehler) with deagglomerated alumina suspension with 0.05 μm particles and washed with triple distilled water (TDW). 0.1 mg of the sialylated trisaccharides were dissolved in 3 mL of 0.1 M KCl. The trisaccharides were electrografted on GCE by applying cyclic voltammetry (CV) in the range of 0.6–1.2 V (vs a Ag/AgCl 3 M KCl reference electrode at a scan rate of 10 mV/s for 5 cycles). The modified electrode was rinsed with TDW and stabilized in 50 mM acetate buffer (pH 5) for 1 h at 37 °C before exposure to the enzyme.

### Preparation of Modified Au Electrodes

AuEs were manually polished on a microcloth pad (Buehler) with deagglomerated alumina suspension with 0.05 μm particles and washed with TDW. Electrodes were deep cast in 1 mL of 1 mM LPA in EtOH for 1 h. The electrodes were rinsed with ethanol. A solution of 1 mg/mL COMU in acetonitrile (ACN) with 1% triethylamine (TEA) was prepared. The electrodes were incubated in the solution for 30 min at 25 °C and washed 2 times with ACN. 0.2 mg of the sialylated trisaccharides were dissolved in 0.2 mL of TDW. The electrodes were drop-cast with 30 μL of the solution for 30 min at 25 °C. The modified electrodes were rinsed with TDW and stabilized in 50 mM acetate buffer (pH 5) for 1 h at 37 °C before exposure to the enzyme.

### Electrochemical Measurements

EIS measurements were performed using a three-electrode standard electrochemical cell with a BioLogic SAS SP-300 potentiostat under single sine AC excitation at a potential of 0.21 V with 10 mV and an amplitude in the frequency range from 100 kHz to 0.1 Hz. The system contains (a) Ag/AgCl (3 M KCl) as the reference electrode, (b) a Pt rod as the counter electrode, and (c) a 3 mm GCE as the working electrode. The measurements were performed in a solution of 5 mM [Fe(CN)_6_]^3–^/[Fe(CN)_6_]^4–^, 100 mM KCl, and 50 mM acetate buffer at pH 5. The results were fitted to the equivalent circuit of *R*_S_[(*R*_CT_*W*)∥*Q*], where *R*_S_ is the resistance of the solution, *R*_CT_ is the charge-transfer resistance of the layer, *Q* is the constant phase element, and *W* is the Warburg diffusion element. Normalized *R*_CT_ is obtained by calculating the ratio between *R*_CT_ after the enzymatic reaction, *R*_CT_ (*t* = *x*), and of the glycan surface before exposure to NA, *R*_CT_ (*t* = 0).

### Exposure to the Enzyme

Stock samples of NA were prepared as described in a previous paper. Stock samples of NA were dissolved in 200 μL of 50 mM acetate Buffer (pH 5). 2 μL of each stock was added to 178 μL of 50 mM acetate buffer, giving a final volume of 0.18 mL (3 mU/mL). Each modified electrode was drop-cast with 50 μL of the solution for 1 h at 37 °C. After the exposure, the electrodes were rinsed with the acetate buffer.

### Exposure to the Enzyme in the Presence of an Antiviral Drug

Stock samples of NA were dissolved in 200 μL of 50 mM acetate buffer (pH 5). 2 μL of each stock was added to 178 μL of a 1 μM antiviral drug in a 50 mM acetate buffer solution, giving a solution final volume of 0.18 mL (3 mU/mL). Each modified electrode was drop-cast with 30 μL of the solution for 1 h. After the reaction, the electrodes were rinsed with acetate buffer.

## Results and Discussion

In our previous study, we developed two platforms modified with a set of four synthetic sialoside trisaccharides for the detection of neuraminidase based on the binding and enzymatic activity of bacterial NA.^23^ The synthetic trisaccharides differ by the regiochemistry of sialic acid and by the type of sialic acid and were equipped with an alkylamine linker.

The first interface is produced by electrografting glassy carbon electrodes (GCEs) with sialosides to give **GCE-H3**, **GCE-H6**, **GCE-M3**, and **GCE-M6** (Figure 2).^23,28,29^ The second interface is produced by coupling amine-terminated trisaccharides to a lipoic acid (LPA) monolayer on a Au electrode (AuE) to give **AuE-H3**, **AuE-H6**, **AuE-M3**, and **AuE-M6** (Figure 2). The glycan monolayers on AuE and GCE surfaces differ in several aspects. The AuE-gla the LPA submonolayer and denser with sialosides compared with GCEs. The sialoside-diluted GCE-glycan surface is more hydrophobic via the exposed glassy carbon.^23^ The eight modified interfaces showed an ability to differentiate impedimetrically between bacterial NA via two distinctive interaction modes. While on AuE, the electrochemical signal was the result of catalytic activity on the sialosides, the response on GCE results from the adhesion of the enzyme to the saccharide-modified layer. We aimed to use the same strategy to discriminate between IV NA enzymes from different strains and compare them to bacterial ones.

**Figure 3:**
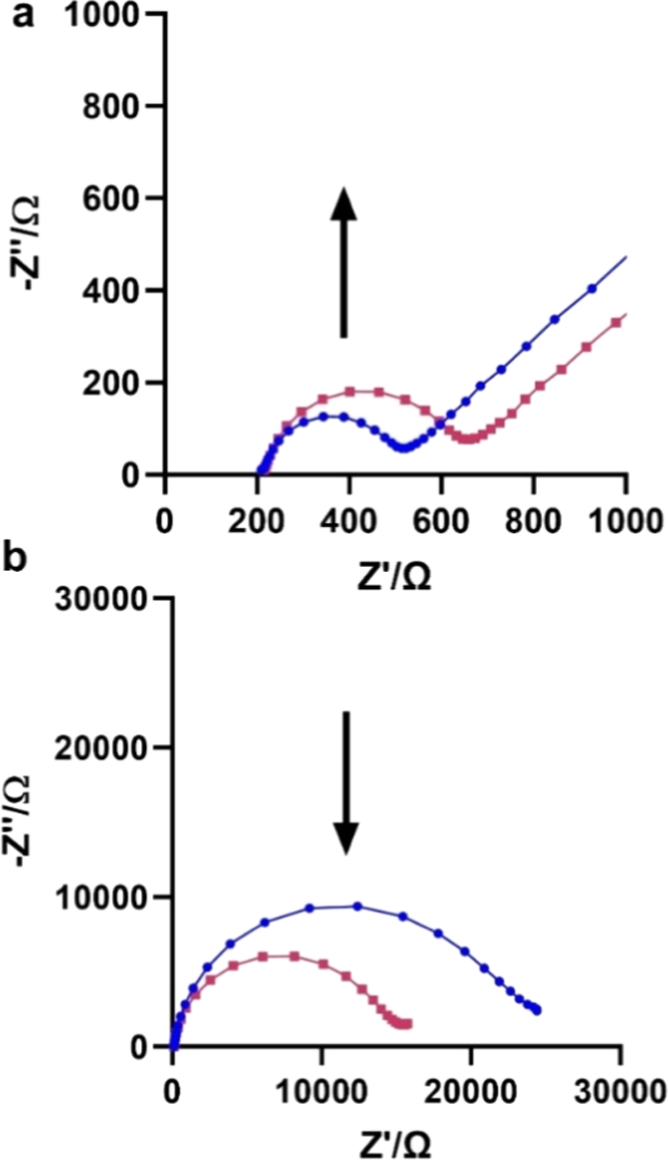
Nyquist plot of response to 3 mU/mL of *H1N1* NA by (a) **GCE-H3**, which shows an increase in impedance, and by (b) **AuE-H3**, which shows decreased impedance. The blue plot is prior to exposure, and the red one is after exposure to the enzyme. The equivalent circuit used is *R*_S_[(*R*_CT_*W*)∥*Q*], where *R*_S_ is the resistance of the solution. *R*_CT_ is the charge-transfer resistance, *W* is the Warburg diffusion element, and *Q* is the constant phase element.

To determine if the systems respond impedimetrically to IV NA in a manner similar to that for bacterial NA, EIS measurement was performed using **GCE-H3** and **AuE-H3** before and after exposure to NA from the *H1N1* virus. The exposure of **GCE-H3** to the enzyme resulted in an increase in charge-transfer resistance (*R*_CT_, Figure 3a), which is in line with our previous observation for NA binding.

The exposure of **AuE-H3** to the enzyme resulted in a decrease of *R*_CT_, which is in line with our previous observation of NA enzymatic activity (Figure 3b). This results from the removal of sialic acid that causes a change in surface charge, dipole, layer size, and density, which increases the ability of the negatively charged redox moiety to penetrate the layer, resulting in a decrease in *R*_CT_. To characterize the interaction of IV NA with glycan-modified gold surfaces, X-ray photoelectron spectroscopy (XPS) and variable angle spectroscopic ellipsometry (VASE) analyses were performed prior to and after exposure to the *H3N2* enzyme. The XPS analyses of the nitrogen/amide region of **Au-H3** show a small peak of N 1s (B.E. 400.1 eV, see Figure S1). Exposure of **Au-H3** to *H3N2* NA did not result in any increase in the N 1s signal. This suggests that no protein was added to the surface. VASE analyses showed an increase in the optical thickness to 12 from 7 Å following the attachment of the trisaccharides to the LPA monolayer. No significant change in the optical thickness was observed following exposure to *H3N2* NA (Figures S3 and S4).

The collective results show that there is no addition of a viral enzyme to the gold surface. This is in accordance with the decrease in the electrochemical impedance, which correlates with the enzymatic reaction. In the case of GCE, XPS analyses performed prior to and after the exposure to the enzyme showed the addition of the protein (Figure S2). However, the increase in the N 1s (B.E. 400.1 eV) signal of viral NA is significantly lower compared to bacterial NA.^23^ This indicates that IV NA has a lower affinity for the saccharide-modified GCE surface, which is in line with a small increase in *R*_CT_.

**Figure 4:**
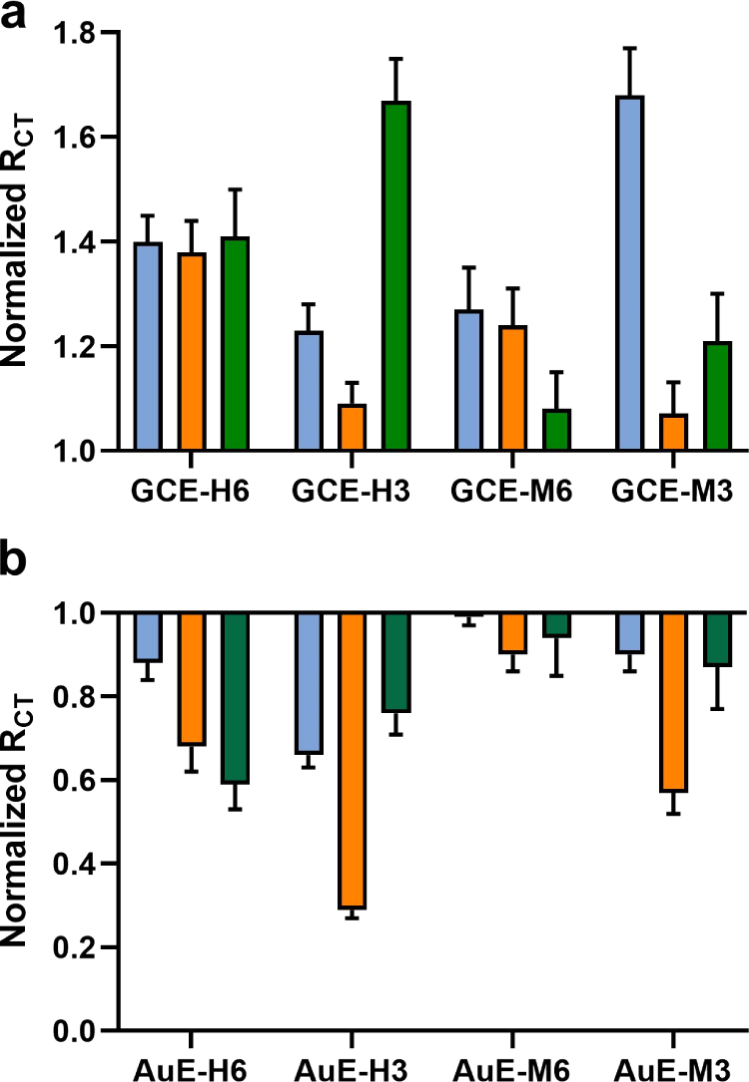
Normalized *R*_CT_ response to *H1N1* (blue), *H3N2* (orange), and *H5N1* (green) NAs by (a) GCE modified with sialosides and (b) AuE modified with sialosides. The standard deviation is based on the response of five electrodes. Normalized *R*_CT_ was calculated by dividing the measured *R*_CT_ after exposure by *R*_CT_ prior to exposure.

To profile IV enzymes from different strains, the eight glycan-modified electrodes were exposed to *H1N1*, *H3N2*, and *H5N1* NAs. The impedimetric response was recorded, and the normalized *R*_CT_ was used to allow a comparison of the different systems (Figure 4). All modified GCEs showed an increase of *R*_CT_ toward the three enzymes. The level of electrochemical impedance increase is both enzyme- and substrate-dependent. *H1N1* NA showed a preference for **GCE**-**M3**, *H5N1* NA showed a preference for **GCE-H3**, and *H3N2* NA showed a slight preference for **GCE-H6** (Figure 4a). It was clear that the GCE system can be selective for *H1N1* and *H5N1* NAs and not for *H3N2* NA. This can result from stronger binding responses *H1N1* and *H5N1* NAs exhibit to saccharide-modified GCEs.

All modified AuEs responded in a decrease of *R*_CT_ toward the three enzymes. The level of electrochemical impedance decreases again, proving to be both enzyme- and substrate-dependent. *H1N1* NA showed a preference for **Au-H3**, *H5N1* NA for **Au-H6**, and *H3N2* NA for **Au-H3** (Figure 4b).

It was clear that the Au system can be selective only for *H3N2* NA. Both *H3N2* and *H1N1* NAs showed the highest activity on **AuE-H3** compared with the other electrodes, which is in line with previous works on influenza NA sialoside preference.^30^ It is noteworthy indicating that *H1N1* NA and *H5N1* NA show high binding to sialosides with α 2–3 linkage and low catalytic activity, while for *H3N2* NA, catalytic activity toward α 2–3 linkages was significant compared to the negligible binding. On the other hand, the α 2–6 linkages did not show any preference for either catalysis or binding.

Although it is clear that there are overlaps in the response of several electrode–sialoside pairs, the combination of the eight pairs gives a distinctive pattern for each enzyme. It is logical to assume that the affinity and catalytic activity of each enzyme toward different sialosides can never be identical. The radar plot analysis takes advantage of the different *R*_CT_ signal intensities to provide a powerful identification tool. As we previously demonstrated, heat maps can also be used to show the differences between enzyme–sialoside preferences.^23^ Heat map presentation allows a detailed comparison by analyzing rows or columns. The radar plot presentation of a multiparametric data set provides a unique fingerprint for each NA, which allows profiling by straightforward shape analysis.

**Figure 5:**
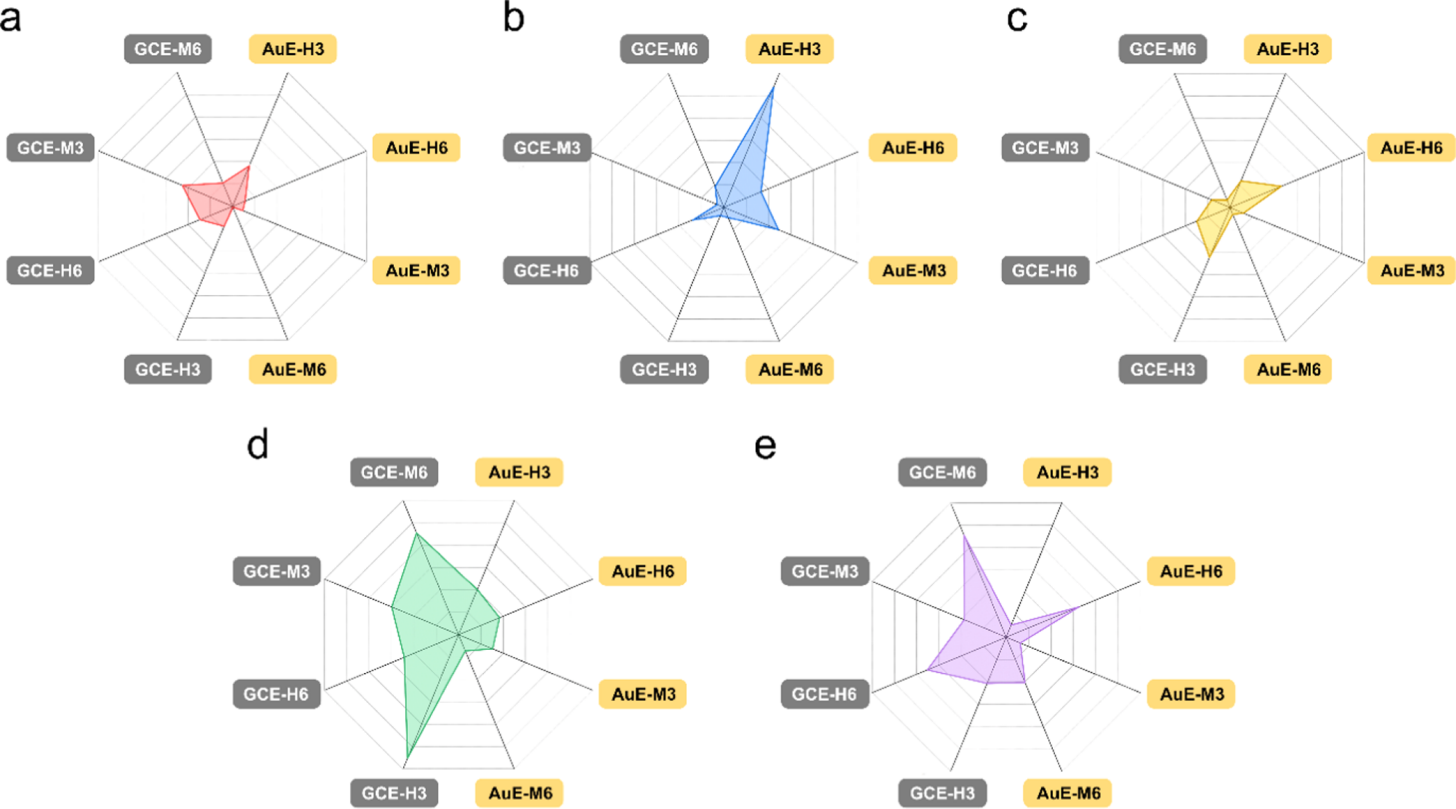
Radar plot of |Log(Normalized *R*_CT_)| for the response of *H1N1* (a), *H3N2* (b), *H5N1* (c), *NACP* (d), and *NAAU* (e) to the presented sensory layers.

In our previous studies, the response of the eight electrodes to bacterial NAs,^12^*Clostridium perfringens* NA (*3NACP*) and *Arthrobacter ureafaciens* NA (*6NAAU*), was studied. To evaluate the characteristic features to distinguish between IV and bacterial NAs, the data are presented in a radar graph (Figure 5d,e). The comparison shows that the bacterial NA has a higher response for binding compared to enzymatic activity. For IV NA, such preference is not as clear, and an even stronger response toward catalysis can be observed in some cases. There are structural differences between IV and bacterial enzymes.

**Figure 6:**
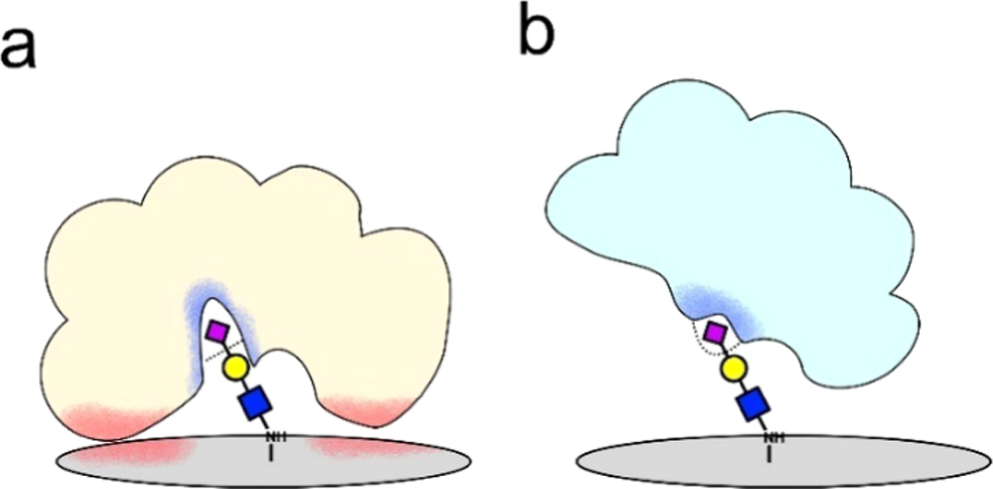
Schematic representation of NA interaction modes on GCE: (a) NA with a deep catalytic site (blue) also experiences hydrophobic interactions between the protein interface and the electrode (red), and (b) NA with a surface-exposed catalytic site (blue) does not experience additional interactions between the protein and the electrode surface.

For bacteria NA, the catalytic pocket is located deeper in the enzyme, and the surrounding hydrophobic interface is exposed to the GCE surface, which can enhance the affinity to the electrode (Figure 6a).^23^ The structure of IV NA (PDBid 7S0I and 4H52 for *H1N1* and *H3N2* NAs, respectively),^31,32^ the catalytic pocket is located close to the surface of the enzyme with low peripheral hydrophobicity, which in turn does not interact with the GCE surface (Figure 6b). Our analysis shows the binding of NA to sialoside-functionalized GCE, and we cannot exclude that in addition to binding, an enzymatic reaction took place. On the other hand, the higher catalytic activity on AuE is combined with the fast detachment of the NA from the glycan-modified Au surface and prevents binding (Figure 6b). The above observations are in line with the XPS analyses that indicate that bacterial NA binds to hydrophobic sialylated GCEs more strongly than the IV ones. The EIS on Au shows a catalytic response of the viral NA, which is supported by VASE analysis.

Glycan arrays rely on fluorescent labeling in colorimetric assays or enhancement via magnetic nanoparticles and microfluidic integrated systems. In microarrays, only one type of interface is used while a huge library of glycans is required to produce sufficient database to produce selectivity.^33,34^ Other methods used for the detection of viral proteins, such as serological essays, suffer from cross-reactivity, resulting in a decrease in strain selectivity.^35^ The electrochemical strategy offers an alternative that overcomes all of the above. The combination of the responses, as manifested in the radar plot, provides NA selectivity. This was achieved by using two types of interfaces, enabling us to double the comparison possibilities using a smaller set of synthetic saccharides. This highlights the advantage of interface modification in selective biosensing.

**Figure 7:**
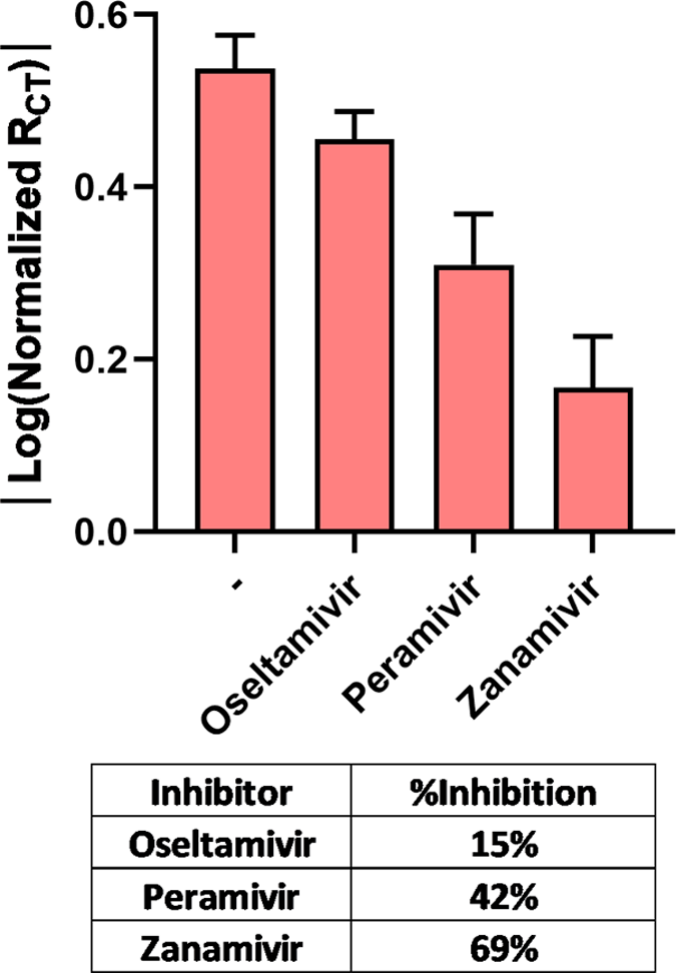
|Log(Normalized *R*_CT_)| response of **AuE-H3** to *H3N2* NA in the presence and absence of viral NA inhibitors. The table contains inhibition %, which was calculated by the equation (Log(*R*_no inhibitor_) – Log(*R*_inhibitor_))/Log(*R*_no inhibitor_). The standard deviation is based on the response of five replicates.

In previous work, impedimetric studies enabled us to assess the decreased bacterial NA catalytic activity on AuE in the presence of an NA inhibitor (Oseltamivir).^23^ Here, we wanted to evaluate the effect of several NA inhibitors on the EIS response to viral NA. The enzymatic response of **AuE-H3** to *H3N2* NA was evaluated in the presence of three known inhibitors, Oseltamivir, Peramivir, and Zanamivir (Figure 7). The results indicate that Oseltamivir had a weak inhibition effect, Peramivir had a moderate inhibition effect, and Zanamivir had the strongest effect. Clinical trials for influenza A show similar trends in inhibition of influenza by these NA inhibitors.^36,37^ Additionally, the physiological conditions of the patient might influence the efficiency of the antiviral drug; hence, the treatment can start with the inhibitor that shows the highest inhibitory activity.^36,37^ It is important to note that the change in ionic strength and the addition of specific metal ions can affect the surface-derived enzyme–substrate interactions.^38^ In our study, we used previously reported conditions, buffers, ionic composition, and strength based on the reactivity evaluation of these enzymes in solution. In the future, further adjustment of these conditions will allow the evaluation of activity and inhibition in a physiological environment. Screening methodologies that utilize PCA often rely on large databases.^10,22^ The above results show that a combination of a biorelevant interaction, e.g., sialoside structure, with an orthogonal surface interaction mode provides a way to rationally increase the database, which can lead to enhanced selectivity.

## Conclusions

Pathogenic neuraminidases bind many types of sialosides, which makes discrimination based on a single glycan analysis impossible. However, binding and catalysis preference toward different sialosides does exist in terms of affinity and hydrolysis kinetics. The ability to characterize each neuraminidase can hence be enabled by profiling their electrochemical response toward a set of sialoside-based electrodes. We designed a system in which each electrode provides three parameters that influence the interaction: sialoside type, regiochemistry, and electrode surface. The developed strategy allows discrimination between NAs and evaluation of inhibitor efficacy. It might be useful both for determining the infection source and for defining the antiviral treatment against different viral strains. Standard analytical techniques rely only on the sialoside structure to profile NAs. We used the unique NA affinity for the surface and submonolayer to provide a glycan-mediated protein–electrode interaction. This provided a new multiparametric electrochemical way to identify each NA, even with the use of a limited set of sialosides. The surface properties and their role in biochemical analysis have generally been overlooked. However, those exact properties can be used to give additional dimension to these interactions. Considering the complexity of glycan synthesis, providing a means to enhance the amount of data without increasing the synthetic load is extremely important.

We demonstrate here that the surface interaction adds useful data that are crucial for characterizing protein families that target similar moieties. This new paradigm in array biosensing suggests that in the future, assembling the same set of receptors on a variety of surfaces will enhance and improve the bioinformatics data.

## Supporting Information Available

Au wafer preparation procedures with their corresponding characterization; and EIS and XPS data (PDF)
