# Supplementary material for: Surface-Controlled Sialoside-Based Biosensing of Viral and Bacterial Neuraminidases
Source: Langmuir. 2024 Mar 30;40(14):7471–8. doi: 10.1021/acs.langmuir.3c03943 (PMC11008233; doi:10.1021/acs.langmuir.3c03943)
Supplement: Supplementary file 1 — la3c03943_si_001.pdf [file la3c03943_si_001.pdf]

Supporting Information

Surface-controlled sialoside-based biosensing of viral and bacterial neuraminidases

Israel Alshanski,[a] Suraj Toraskar, [b] Daniel Gordon-Levitan,[a] Marco Massetti,[a,c] Prashant Jain,[b] Luigi Vaccaro,[c] Raghavendra Kikkeri,[b*] Mattan Hurevich,[a*] Shlomo Yitzchaik[a*]

[a] The institute of chemistry and Center of Nanotechnology, The Hebrew University of Jerusalem. Jerusalem, 91904, Israel.

[b] Indian Institute of Science Education and Research, Dr. Homi Bhabha Road, Pune-411008, India.

[c] Laboratory of Green Synthetic Organic Chemistry Dipartimento di Chimica, Biologiae Biotecnologie Università di Perugia, Via Elce di Sotto 8, 06123 Perugia Italy.

Contents

Methods ... 3
Surface modifications and characterizations ... 3
Glycoside structure ... 4
XPS and electrochemical results ... 4
XPS Analyses ... 4
Variable angle spectroscopic ellipsometry (VASE) results ... 6
Nyquist Plot of GCE sialosides with H3N2 neuraminidase: ... 7
Nyquist Plot of GCE sialosides with H1N1 neuraminidase: ... 9
Nyquist Plot of GCE sialosides with H5N1 neuraminidase: ... 11
Nyquist Plot of AuE-sialosides with H3N2 neuraminidase: ... 13
Nyquist Plot of AuE-sialosides with H1N1 neuraminidase: ... 15
Nyquist Plot of AuE-sialosides with H5N1 neuraminidase: ... 17
Nyquist Plot of AuE-sialosides with H3N2 neuraminidase and Inhibitors: ... 19

Methods

All synthetic procedures, electrochemical and surface analyses were performed by the same protocols that were reported in our previous work (https://doi.org/10.1021/acschembio.2c00913).

Surface modifications and characterizations

Preparation of modified Au Surfaces. Modified Au surfaces with LPA were prepared by previously reported protocol. The saccharide was coupled to the surface by same protocol used for modification of AuE. Exposure to enzyme performed by same method as described for GCE. Substrates were Rinsed with TDW and dried with nitrogen before measurements.

Characterization of modified Au surface. Surface characterizations of modified Au surfaces were performed by X-ray photoelectron spectroscopy (XPS) and variable angle spectroscopic ellipsometry (VASE). XPS measurements were performed using Axis Supra+ spectrometer (Kratos Analytical Ltd., Manchester, U.K.) with Al Kα monochromatic x-ray source (1486.7 eV). The XPS spectra were acquired with a takeoff angle of 90° (normal to analyzer); vacuum condition in the chamber was 1.9 nTorr. High-resolution XPS spectra were acquired with a pass energy of 20 eV and step size of 0.1 eV. The binding energies were calibrated according to the C 1s peak position (285.0 eV). Data were collected and analyzed by using Casa XPS (Casa Software Ltd.) and Vision data processing program (Kratos Analytical Ltd.). Surface characterizations of modified Au surfaces were performed by the same methods as described for GCP. Variable angle spectroscopic ellipsometry (VASE) analyses were performed using VB-400 ellipsometer (Woollam Co.) at Brewster angle of 75° scanning from 300 to 900 nm wavelength and fitting with Cauchy model on gold surface retaining relationship of $n(\lambda) = A + \frac{B}{\lambda^2} + \frac{C}{\lambda^2}$ where B =0.1 and C= 0.01 (full model detail can be found in: https://doi.org/10.1006/rwsp.2000.0070). See also: “*Ellipsometry and Polarized Light*” by R.M.A. Azzam & N.M. Bashara, North Holland Personal Library; Elsevier Science B.V., 1996.

Glycoside structure

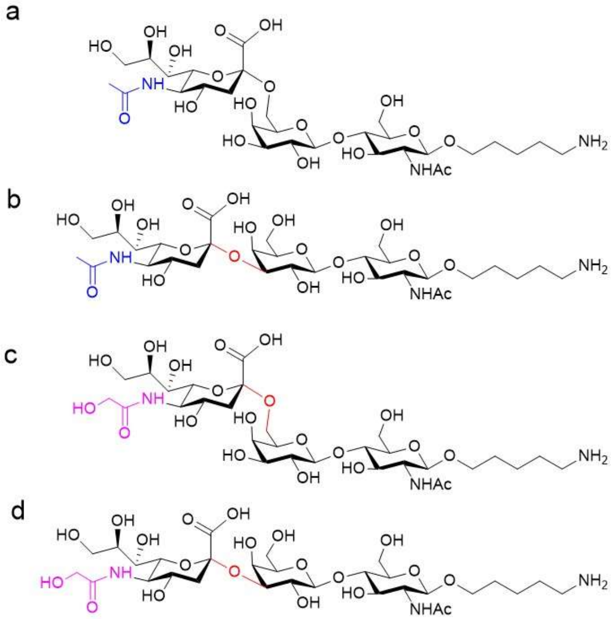

Scheme S1: Sialoside structures used in the study for H3 (a), H6 (b), M3 (c), and M6(d) where in red is the sialic acid connectivity, in blue the acetamide of Neu5Ac, and in purple the glycolylamide of Neu5Gc.

XPS and electrochemical results

XPS Analyses

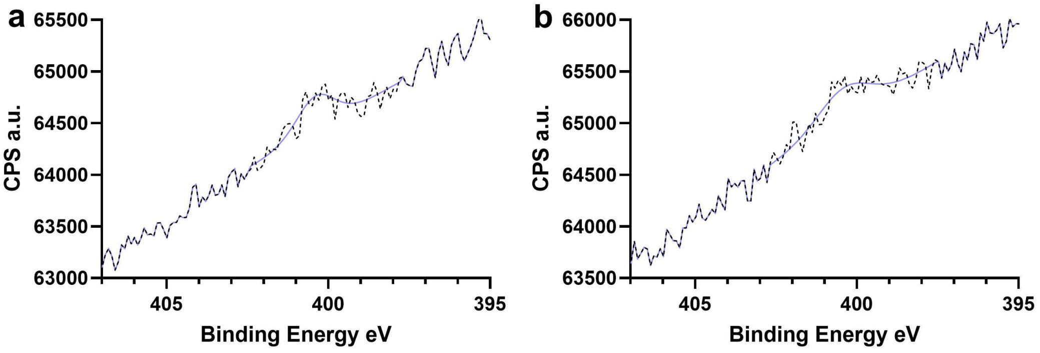

Figure S1: XPS analyses of N1s for Au-H3 prior (a) and after (b) exposure to neuraminidase H3N2 with peak at 400.1 corresponding to N1s of amide of the sialoside without addition of amide corresponding to the presence of protein on the surface.

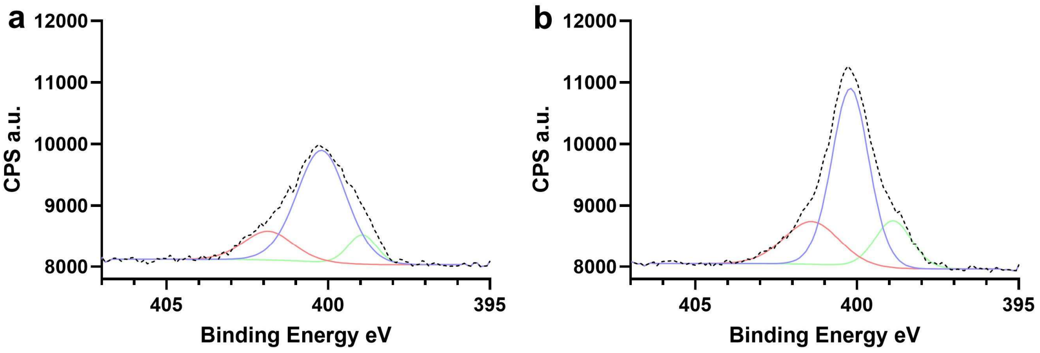

Figure S2: XPS analyses of N1S for GCE-H3 prior (a) and after (b) exposure to neuraminidase H3N2. Where red correlates with ammonium, blue with amide, and green with nitride. Increase of amide on GCE at peak of 400.1 (Blue) corresponds to attachment of protein to the surface.

Variable angle spectroscopic ellipsometry (VASE) results

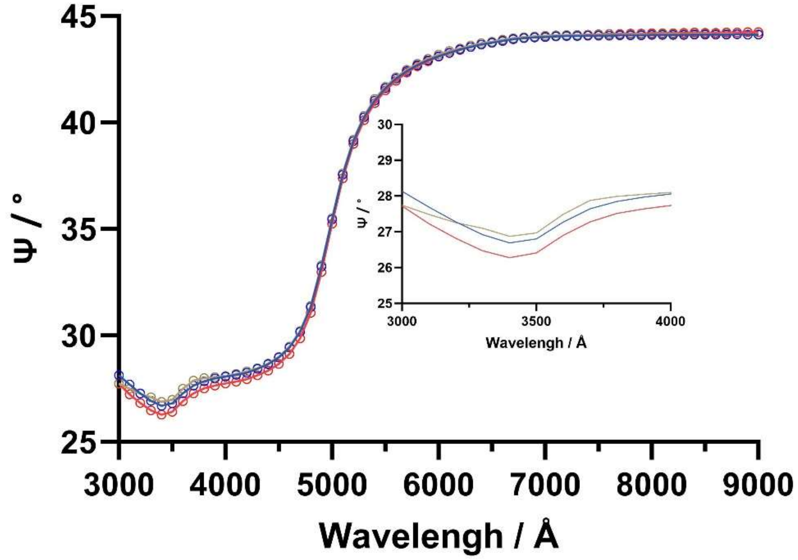

Figure S3: Psi of VASE analyses of Au-LPA (Red) and Au-LPA-H3 prior (Blue) and after (Brown) exposure to neuraminidase H3N2 where the line is fit and the circles are raw data. Insert contains zoom to the region with the largest difference.

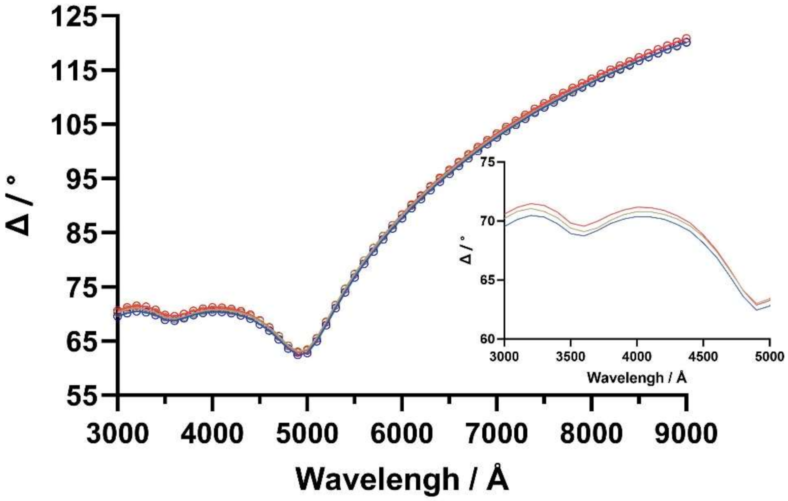

Figure S4: Delta of VASE analyses of Au-LPA (Red) and Au-LPA-H3 prior (Blue) and after (Brown) exposure to neuraminidase H3N2 where the line is fit and the circles are raw data. Insert contains zoom to the region with the largest difference.

Nyquist Plot of GCE sialosides with H3N2 neuraminidase:

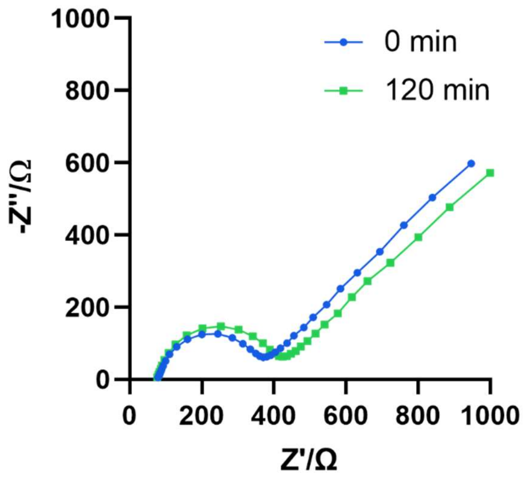

Figure S5: Nyquist plot of GCE-H3 with H3N2 neuraminidase before (blue) and after (green) exposure.

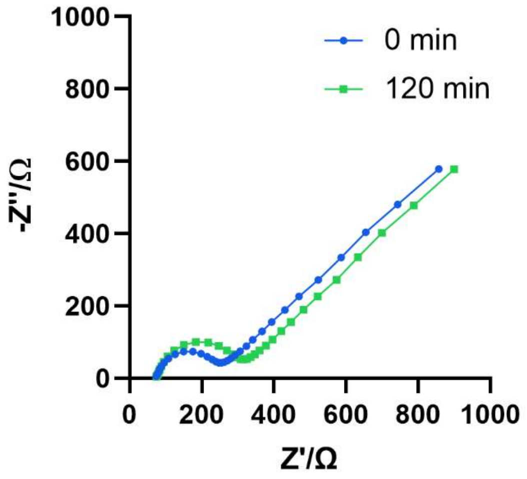

Figure S6: Nyquist plot of GCE-H6 with H3N2 neuraminidase before (blue) and after (green) exposure.

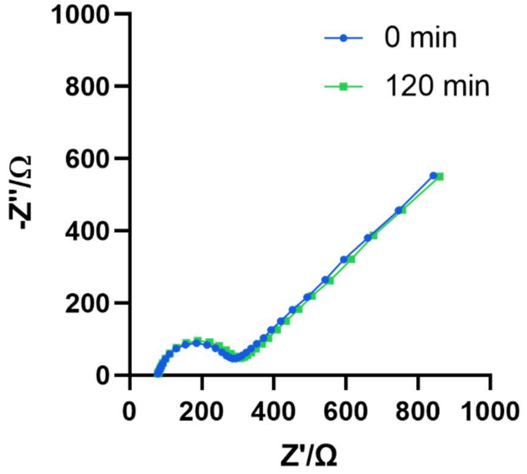

Figure S7: Nyquist plot of GCE-M3 with H3N2 neuraminidase before (blue) and after (green) exposure.

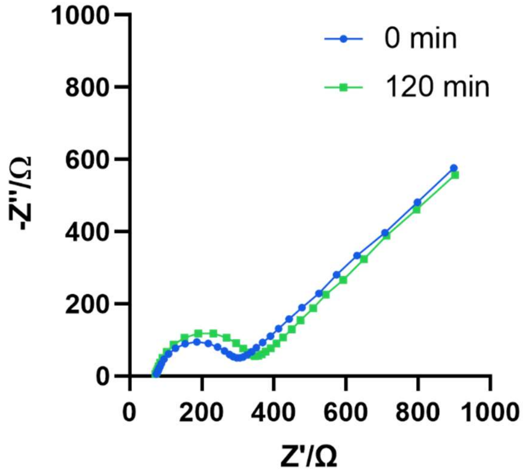

Figure S8: Nyquist plot of GCE-M6 with H3N2 neuraminidase before (blue) and after (green) exposure.

Nyquist Plot of GCE sialosides with H1N1 neuraminidase:

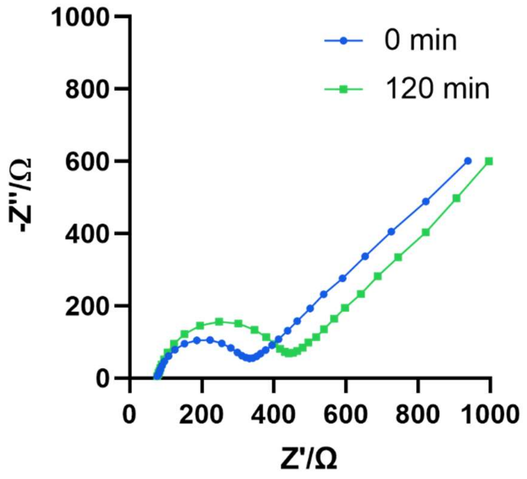

Figure S9: Nyquist plot of GCE-H6 with H1N1 neuraminidase before (blue) and after (green) exposure.

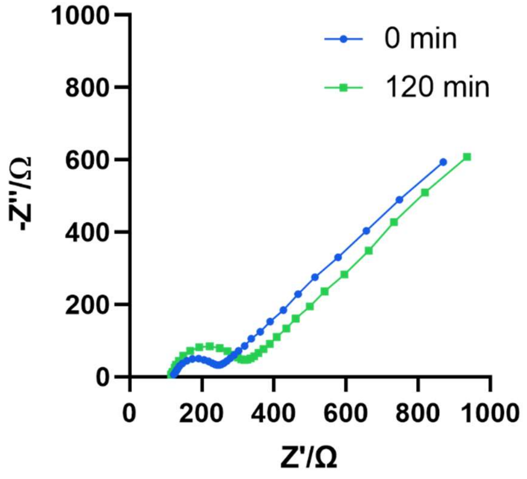

Figure S10: Nyquist plot of GCE-M3 with H1N1 neuraminidase before (blue) and after (green) exposure.

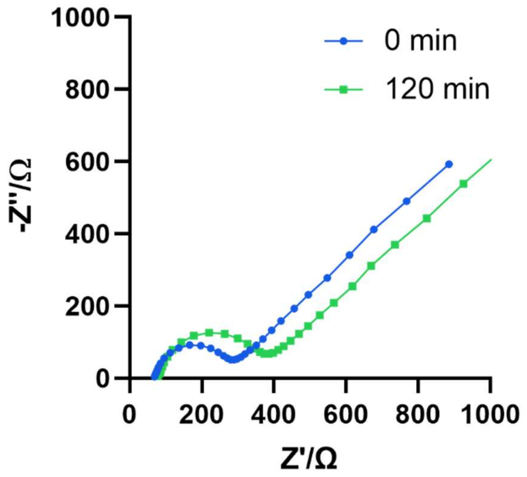

Figure S11: Nyquist plot of GCE-M6 with H1N1 neuraminidase before (blue) and after (green) exposure.

Nyquist Plot of GCE sialosides with H5N1 neuraminidase:

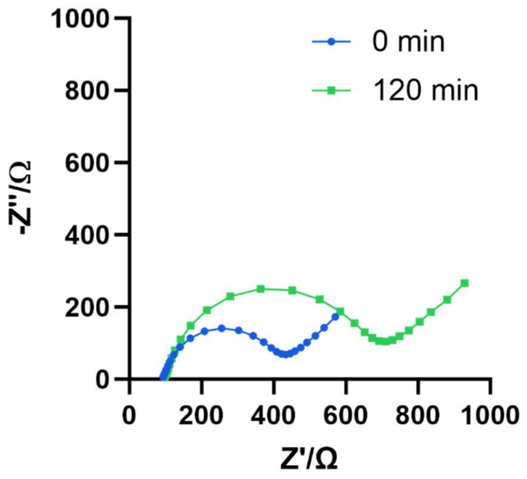

Figure S12: Nyquist plot of GCE-H3 with H5N1 neuraminidase before (blue) and after (green) exposure.

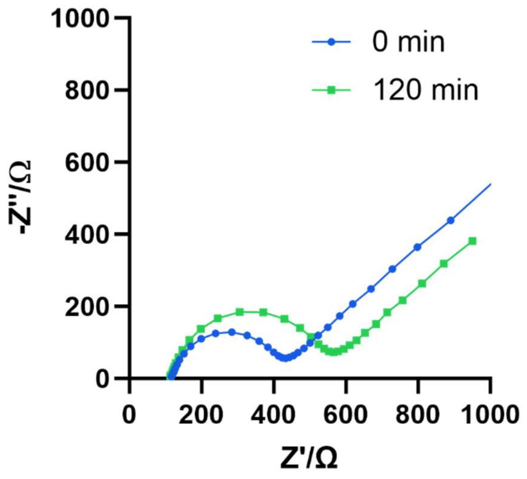

Figure S13: Nyquist plot of GCE-H6 with H5N1 neuraminidase before (blue) and after (green) exposure.

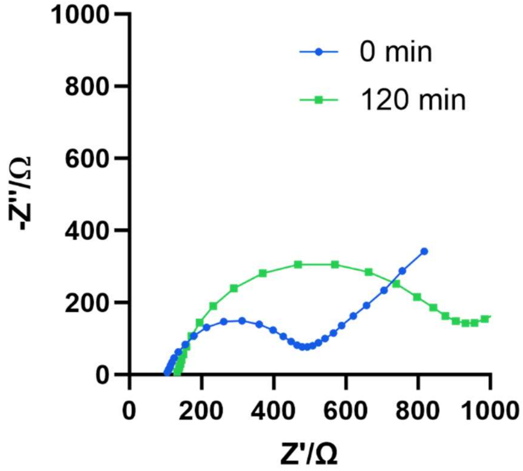

Figure S14: Nyquist plot of GCE-M3 with H5N1 neuraminidase before (blue) and after (green) exposure.

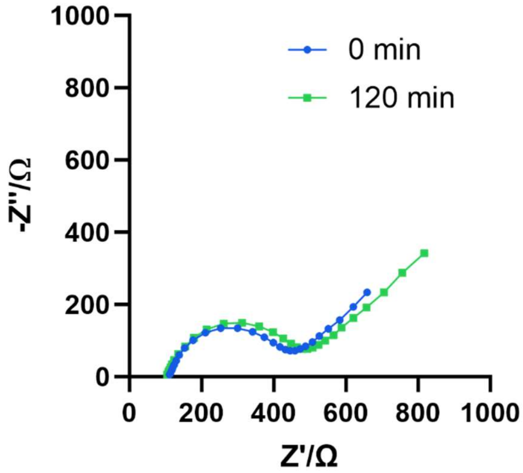

Figure S15 Nyquist plot of GCE-M6 with H5N1 neuraminidase before (blue) and after (green) exposure.

Nyquist Plot of AuE-sialosides with H3N2 neuraminidase:

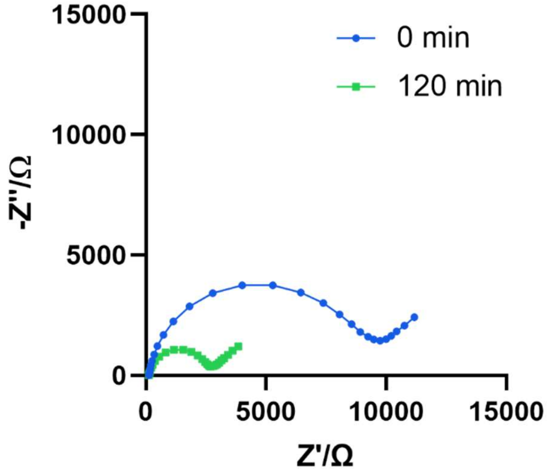

Figure S16: Nyquist plot of AuE-H3 with H3N2 neuraminidase before (blue) and after (green) exposure.

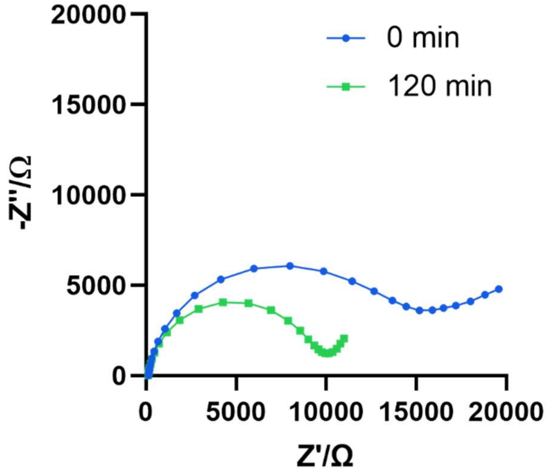

Figure S17: Nyquist plot of AuE-H6 with H3N2 neuraminidase before (blue) and after (green) exposure.

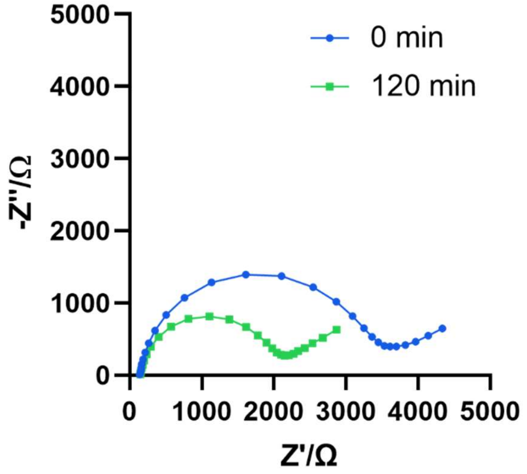

Figure S18: Nyquist plot of AuE-M3 with H3N2 neuraminidase before (blue) and after (green) exposure.

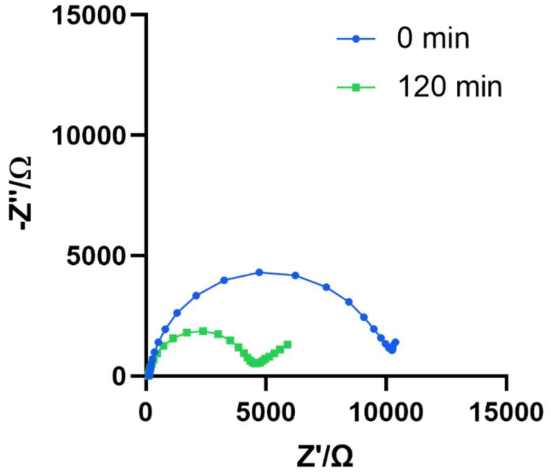

Figure S19: Nyquist plot of AuE-M6 with H3N2 neuraminidase before (blue) and after (green) exposure.

Nyquist Plot of AuE-sialosides with H1N1 neuraminidase:

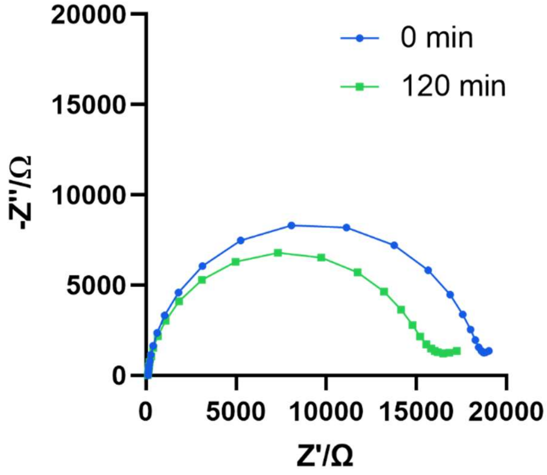

Figure S20: Nyquist plot of AuE-H6 with H1N1 neuraminidase before (blue) and after (green) exposure.

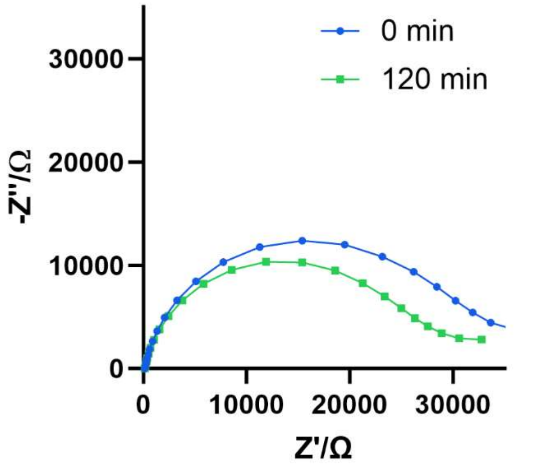

Figure S21: Nyquist plot of AuE-M3 with H1N1 neuraminidase before (blue) and after (green) exposure.

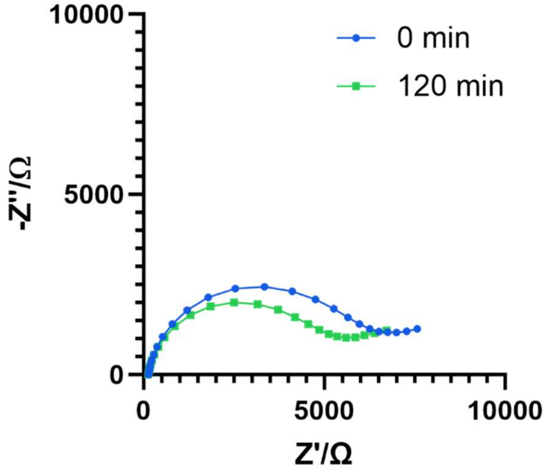

Figure S22: Nyquist plot of AuE-M6 with H1N1 neuraminidase before (blue) and after (green) exposure.

Nyquist Plot of AuE-sialosides with H5N1 neuraminidase:

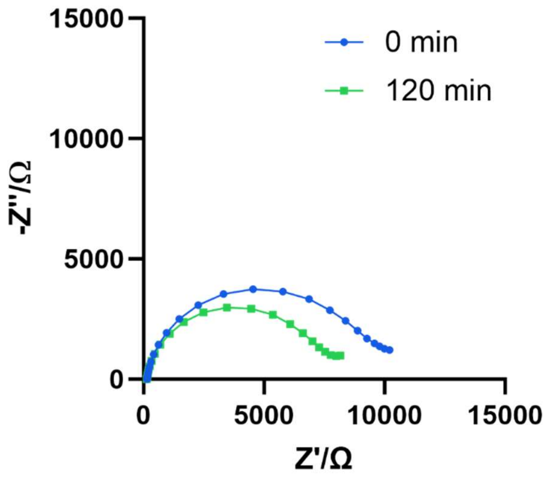

Figure S23: Nyquist plot of AuE-H3 with H5N1 neuraminidase before (blue) and after (green) exposure.

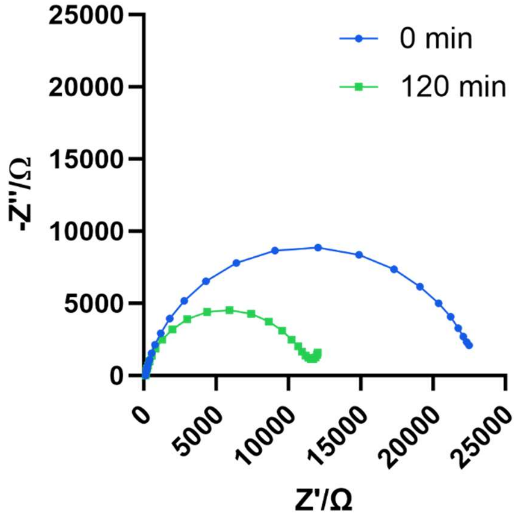

Figure S24: Nyquist plot of AuE-H6 with H5N1 neuraminidase before (blue) and after (green) exposure.

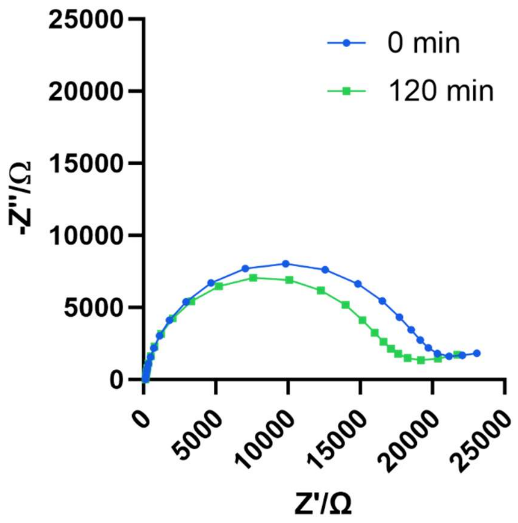

Figure S25: Nyquist plot of AuE-M3 with H5N1 neuraminidase before (blue) and after (green) exposure.

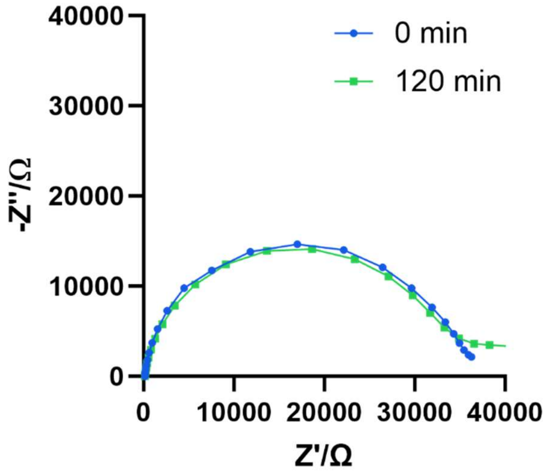

Figure S26: Nyquist plot of AuE-M6 with H5N1 neuraminidase before (blue) and after (green) exposure.

Nyquist Plot of AuE-sialosides with H3N2 neuraminidase and Inhibitors:

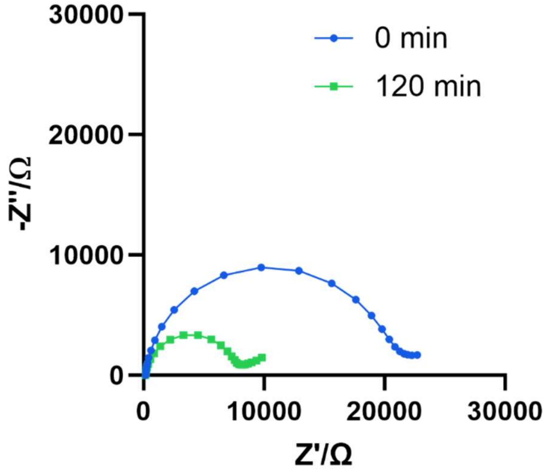

Figure S27: Nyquist plot of AuE-H3 with H3N2 neuraminidase with presence of oseltamivir before (blue) and after (green) exposure.

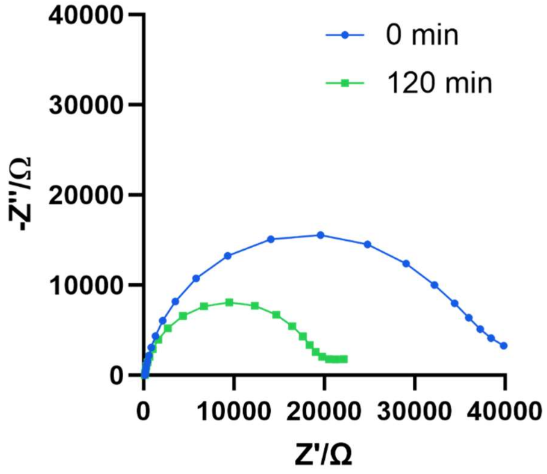

Figure S28: Nyquist plot of AuE-H3 with H3N2 neuraminidase with presence of zanamivir before (blue) and after (green) exposure.

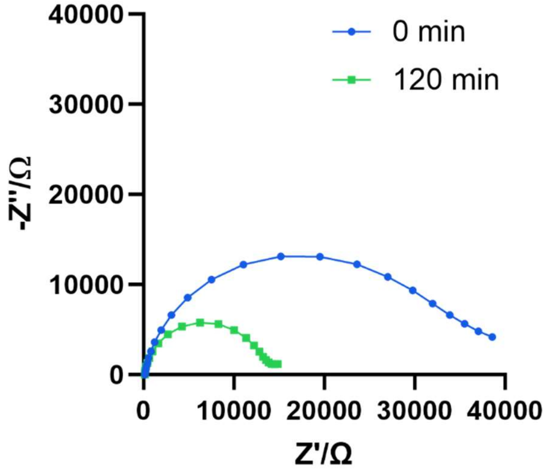

Figure S29: Nyquist plot of AuE-H3 with H3N2 neuraminidase with presence of peramivir before (blue) and after (green) exposure.
